# Blood Clots versus PRF: Activating TGF-β Signaling and Inhibiting Inflammation In Vitro

**DOI:** 10.3390/ijms23115897

**Published:** 2022-05-24

**Authors:** Zahra Kargarpour, Layla Panahipour, Richard J. Miron, Reinhard Gruber

**Affiliations:** 1 Department of Oral Biology, University Clinic of Dentistry, Medical University of Vienna, 1090 Vienna, Austria; zahra.kargarpooresfahani@meduniwien.ac.at (Z.K.); layla.panahipour@meduniwien.ac.at (L.P.); 2 Department of Periodontology, School of Dental Medicine, University of Bern, 3012 Bern, Switzerland; richard.miron@zmk.unibe.ch

**Keywords:** platelet-rich fibrin, blood clot, inflammation, TGF-β, toll-like receptors

## Abstract

The preparation of platelet-rich fibrin (PRF) requires blood centrifugation to separate the yellow plasma from the red erythrocyte fraction. PRF membranes prepared from coagulated yellow plasma are then transferred to the defect sites to support tissue regeneration. During natural wound healing, however, it is the unfractionated blood clot (UBC) that fills the defect site. It is unclear whether centrifugation is necessary to prepare a blood-derived matrix that supports tissue regeneration. The aim of the present study was to compare lysates prepared from PRF and UBC based on bioassays and degradation of the respective membranes. We report here that lysates prepared from PRF and UBC membranes similarly activate TGF-β signaling, as indicated by the expression of interleukin 11 (IL-11), NADPH oxidase 4 (NOX-4) and proteoglycan 4 (PRG4) in gingival fibroblasts. Consistently, PRF and UBC lysates stimulated the phosphorylation and nuclear translocation of Smad3 in gingival fibroblasts. We further observed that PRF and UBC lysates have comparable anti-inflammatory activity, as shown by the reduction in lipopolysaccharide (LPS)-induced IL-6, inducible nitric oxidase synthase (iNOS) and cyclooxygenase 2 (COX-2) expression in RAW264.7 cells. Moreover, inflammation induced by Poly (1:C) HMW and FSL-1, which are agonists of Toll-like receptor (TLR) 3 and 2/6, respectively, was reduced by both PRF and UBC. PRF and UBC lysates reduced the nuclear translocation of p65 in LPS-induced RAW264.7 cells. In contrast to the similar activity observed in the bioassays, UBC membranes lack the structural integrity of PRF membranes, as indicated by the rapid and spontaneous disintegration of UBC membranes. We show here that the lysates prepared from PRF and UBC possess robust TGF-β and anti-inflammatory activity. However, visual inspection of the PRF and UBC membranes confirmed the negative impact of erythrocytes on the structural integrity of membranes prepared from whole blood. The data from the present study suggest that although both UBC and PRF have potent TGF-β and anti-inflammatory activity, UBC does not have the strength properties required to be used clinically to prepare applicable membranes. Thus, centrifugation is necessary to generate durable and clinically applicable blood-derived membranes.

## 1. Introduction

Platelet-rich fibrin (PRF) membranes are generated following the centrifugation of venous blood, which stimulates blood coagulation, followed by compression of the yellow clot to squeeze out the serum [1]. PRF membranes were first introduced in 2001 as a natural source of various blood-derived growth factors that have been shown to promote wound healing [1,2]. PRF membranes are widely used in regenerative dentistry for alveolar ridge preservation following tooth extraction [3], increasing keratinized mucosa at the implant peripheral sites [4], bone augmentation by preparing sticky bones [5], periodontal therapy [6,7], and reducing the side effects of third molar surgery, such as pain and swelling caused by inflammation [8]. In vitro studies support the clinical advantages of PRF, indicating that lysates of PRF membranes and liquid PRF can reduce LPS-induced inflammation in murine macrophages and suppress osteoclastogenesis [9,10,11]. Moreover, PRF is rich in growth factors that are embedded in the fibrin-rich membrane [12]. TGF-β is the central growth factor in PRF [13,14]. TGF-β is a strong inducer of the target genes IL-11, NOX-4 and PRG4 in gingival fibroblasts [13]. Thus, in vitro anti-inflammatory effects and TGF-β activity can both serve as biomarkers to evaluate the activity of PRF membranes and identify the activity of blood clots and membranes made thereof.

Blood clotting, which is similar to what occurs in blood collection tubes to prepare PRF, rapidly occurs at defect sites and thereby initiates the early stages of natural wound healing [15]. In contrast to PRF, which consists of the fibrin-rich matrix enriched in platelets and leucocytes, the natural blood clot additionally entraps erythrocytes, which are removed by centrifugation when preparing PRF. However, all growth factors and bioactive molecules released by activated platelets and leucocytes, as well as the components of the fibrin-rich matrix of coagulated plasma, are present in the blood clot, similar to PRF [12]. Considering the impact of differential centrifugation on the preparation of PRF membranes, there is a moderate but relative increase in platelets and leucocytes in PRF compared to those in natural blood clots [16,17]. One can argue that PRF membranes are better than blood clots because the growth factors and other bioactive molecules are enriched in PRF compared to the unfractionated blood clot. However, this assumption remains a hypothesis, as cellular responses to lysates of PRF membranes and blood clots have not been directly compared in vitro.

Investigations on the bioactivity of lysates prepared from PRF and unfractionated natural blood clot membranes based on our established in vitro anti-inflammatory [9,11,18] and TGF-β activity assays [13,19,20] have yet to be conducted. A previous study by our group observed minimal impact of the centrifugation protocol on in vitro anti-inflammatory and TGF-β activity in solid PRF membranes, suggesting that the impact of blood fractionation on the cellular activity of PRF lysates is low [18]. To better address this question, the in vitro anti-inflammatory and TGF-β activity but also the biomechanical properties of the PRF membrane, which is an essential characteristic of the membranes, can be examined [19,20]. The biomechanical properties of the membranes are likely influenced by the presence of erythrocytes. Erythrocytes change the rheological properties of the blood clot; for instance, volume shrinkage in the clot occurs when activated platelets pull on the fibrin network [21]. This shrinkage occurs due to the compaction of erythrocytes to the center of the clot and the redistribution of platelets and fibrin toward the exterior site of the clot [21]. Erythrocytes can also regulate thrombin and fibrin formation, impacting the viscoelastic properties of the blood clot [21]. The viscoelasticity of plasma clots without erythrocytes increased significantly compared to that of clots with a high amount of erythrocytes [22]. Overall, erythrocytes are incorporated into the spaces between fibers, where they can interrupt the fibrin network structure. Thus, the presence of erythrocytes presumably lowers the biomechanical properties of the membranes prepared from an unfractionated blood clot, but this clinically relevant aspect needs further investigation.

Considering that whole blood is the origin of growth factors and cells, it is unclear whether membranes prepared from an unfractionated blood clot (UBC) have the same effect on TGF-β activation or reduce inflammation as PRF membranes. No attempts have previously been made to investigate the necessity of the centrifugation step to promote the activity of growth factors and cells that exist in whole blood. Moreover, although plasma is used in many in vitro fibrin investigations, erythrocytes (red blood cells) are found in clots and thrombi in vivo. Therefore, this research was conducted to identify the impact of centrifugation on the cellular activity of lysates extracted from both PRF and UBC membranes and to further compare their structural integrity. This study will help clinicians gain insight into the necessity of centrifugation during PRF preparation.

## 2. Results

### 2.1. Loss of Integrity in Membranes Prepared from Unfractionated Blood Clot (UBC)

**Figure 1:**
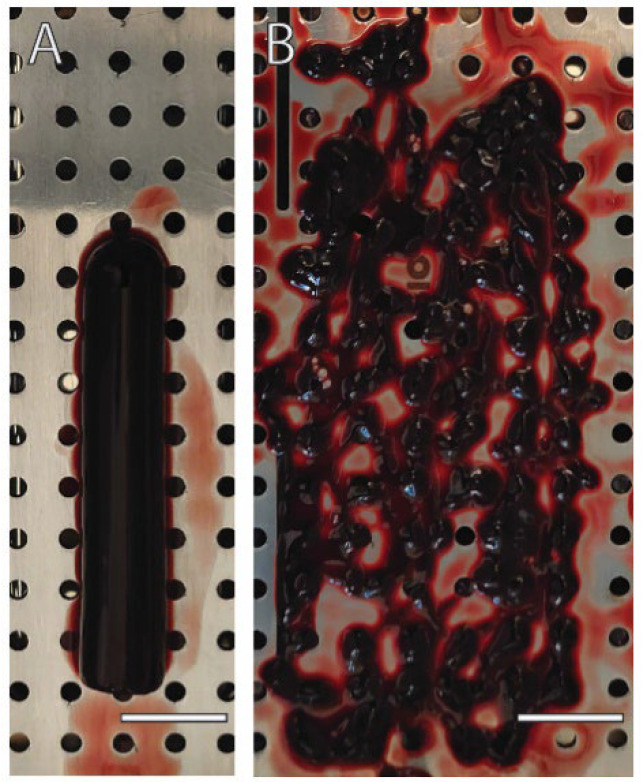
UBC membranes are fragments lacking clinically relevant biomechanical properties. (**A**) Coagulated unfractionated blood forms rods that could be pulled from the plain glass tubes. (**B**) Compression of the UBC on a porous steel plate failed to generate mechanically stable membranes. The pseudo UBC membranes appear as fragments lacking the integrity of a clinically relevant PRF membrane. However, the fragments of the UBC membranes could be harvested and further used in bioassays of TGF-β and anti-inflammatory activity. Scale bars indicate 12 mm.

First, we prepared a red clot from unfractionated blood using plain glass tubes to generate solid PRF. The blood turned into a red rod that could be easily pulled from the tubes without the help of forceps and placed onto the compression device that is used in the clinic to prepare PRF. Compression was performed by placing a steel lid on the UBC to squeeze out the serum, similar to what is observed when preparing PRF membranes. In contrast to that of PRF membranes, however, the integrity of UBC membranes was disturbed and exhibited with an irregular surface that was characterized by holes in a disintegrated compressed red clot (Figure 1). It was impossible to lift the UBC membranes to perform biomechanical tests. It was, however, possible to use the disintegrated compressed UBC membranes to prepare lysates that could be further used for bioassays, similar to those prepared from PRF membranes.

### 2.2. Lysates from PRF and UBC Increased TGF-β Target Genes in Gingival Fibroblasts

**Figure 2:**
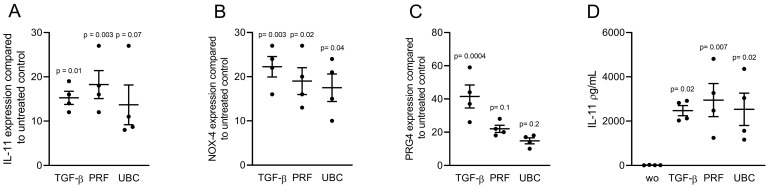
Lysates from PRF and UBC increase TGF-β target genes in gingival fibroblasts. Gingival fibroblasts were incubated with PRF and UBC lysates, and TGF-β was used as a positive control. (**A**–**C**) Real Time-PCR analysis of IL-11, NOX-4 and PRG4. (**D**) Quantification of IL-11 levels in the supernatant by immunoassay. *n* = 4. Statistical analysis was based on uncorrected Dunn’s tests, and *p* values are indicated compared to the untreated control. Significance was set at *p* < 0.05.

To investigate the effect of blood centrifugation on TGF-β activity, gingival fibroblasts were exposed to 30% lysates generated from PRF and UBC membranes. TGF-β (10 ng/mL) served as a positive control. Changes in gene expression were analyzed and revealed that IL-11, NOX-4, and PRG4 were similarly upregulated in the presence of lysates prepared from PRF and UBC (Figure 2A–C). In support of the gene expression data, lysates prepared from PRF and UBC increased the protein levels of IL-11 in gingival fibroblasts (Figure 2D). These observations suggest that both PRF and UBC lysates produced by freeze–thawing and sonication, when normalized to the weight of the respective membranes, can activate TGF-β target genes.

### 2.3. Lysates from PRF and UBC Activated Smad Signaling in Gingival Fibroblasts

**Figure 3:**
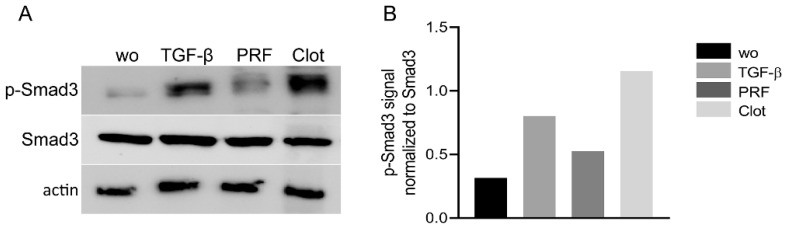
PRF and UBC lysates activated the phosphorylation of Smad3. (**A**) Gingival fibroblasts were exposed to 30% PRF or UBC lysate and TGF-β for one hour following overnight serum starvation. Western blot analysis showed a clear increase in basal Smad3 phosphorylation in response to both PRF and UBC lysates. (**B**) Data indicate the relative changes normalized to Smad3; “wo” indicates without and represents unstimulated cells.

**Figure 4:**
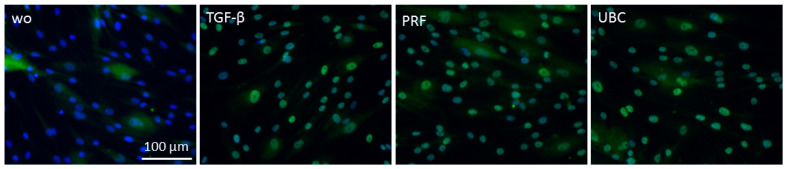
PRF lysate activated the translocation of Smad2/3 from the cytoplasm into the nucleus. Gingival fibroblasts were exposed to TGF-β as a positive control and PRF and UBC lysates. Immunofluorescence analysis of the nuclear translocation of Smad2/3 is shown. The blue dots represent nuclei which is hidden by the green dots representing antibody-positive nuclei; “wo” indicates without and represents unstimulated cells. Microscopic magnificence was set at 20×.

As canonical TGF-β activity acts via Smad proteins [23], we examined whether PRF and UBC lysates trigger the nuclear translocation and phosphorylation of Smad3. To investigate the effect of PRF and UBC lysates on the activation of Smad signaling, Western blot analysis of p-Smad3 was carried out (Figure 3). The results showed that PRF and UBC lysates could intensify Smad3 phosphorylation. To further confirm TGF-β signaling activity, the nuclear translocation of Smad2/3 was investigated (Figure 4). Consistent with the previous data, immunofluorescence analysis revealed that both PRF and UBC lysates could induce the nuclear translocation of Smad2/3 (98%). Together, these findings indicate that both PRF and UBC lysates could activate the canonical TGF-β pathway in gingival fibroblasts.

### 2.4. Lysate from PRF and UBC Reduced LPS-Induced Inflammation in RAW264.7 Cells

**Figure 5:**
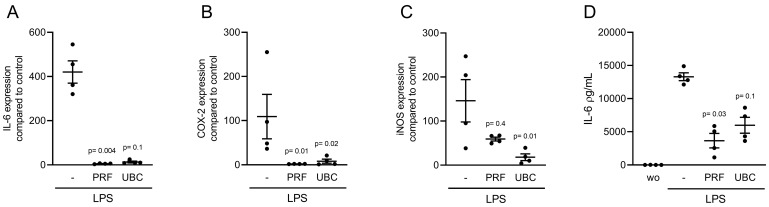
Lysate from PRF and UBC can reduce LPS-induced inflammation in RAW264.7 cells. RAW264.7 cells were exposed to 30% lysates extracted from PRF and UBC membranes in the presence of 100 ng/mL LPS. (**A**–**C**) The data show the x-fold changes in IL-6, COX-2 and iNOS gene expression (**D**) and IL-6 levels in the cell supernatant, *n* = 4. Statistical analysis was based on uncorrected Dunn’s tests, and *p* values are indicated compared to the LPS group. Significance was set at *p* < 0.05.

To investigate the anti-inflammatory effect of PRF and UBC lysates, RAW264.7 cells were incubated with 100 ng/mL LPS in the presence or absence of 30% lysates extracted from PRF and UBC. Analysis of inflammatory marker gene expression indicated that IL-6, COX-2 and iNOS were downregulated in the presence of lysates prepared from PRF and UBC (Figure 5A–C). To confirm these findings, we measured the levels of IL-6 protein in the supernatant of RAW264.7 cells. Consistent with the gene expression data, lysates prepared from PRF and UBC decreased IL-6 protein levels in RAW264.7 cells (Figure 5D). Together, these data showed that lysates extracted from PRF or UBC could reduce LPS-induced inflammation in macrophages.

### 2.5. Lysates Extracted from PRF or UBC Reduced TLR Agonist-Induced Inflammation in RAW264.7 Cells

**Figure 6:**
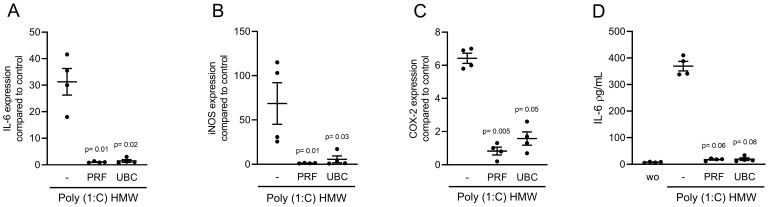
Lysates extracted from PRF or UBC can reduce TLR3 agonist-induced inflammation in RAW264.7 cells. RAW264.7 cells were exposed to 30% lysates extracted from PRF and UBC membranes in the presence of 10 µg/mL poly (1:C) HMW. (**A**–**C**) The data show the x-fold changes in IL-6, COX-2, and iNOS gene expression (**D**) and IL-6 levels in the cell supernatant, *n* = 4. Statistical analysis was based on uncorrected Dunn’s tests, and *p* values are indicated compared to the poly (1:C) HMW group. Significance was set at *p* < 0.05.

**Figure 7:**
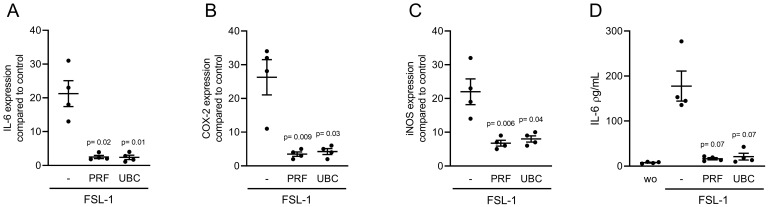
Lysates extracted from PRF or UBC can reduce TLR2/6 agonist-induced inflammation in RAW264.7 Cells. RAW264.7 cells were exposed to 30% lysates extracted from PRF and UBC membranes in the presence of 10 µg/mL FSL-1. (**A**–**C**) The data show the x-fold changes in IL-6, COX-2 and iNOS gene expression (**D**) and IL-6 levels in the cell supernatant, *n* = 4. Statistical analysis was based on uncorrected Dunn’s tests, and *p* values are indicated compared to the FSL-1 group. Significance was set at *p* < 0.05.

To understand whether the anti-inflammatory activity of PRF and UBC lysates was limited to the pathway activated by LPS through TLR4 [24], we induced an inflammatory response in RAW264.7 cells with Poly (1:C) HMW and FSL-1, which are agonists of TLR3 and TLR2/6, respectively. In the presence of Poly (1:C) HMW and FSL-1, both PRF and UBC lysates could reduce the expression of IL-6, COX-2 and iNOS in RAW264.7 cells (Figure 6 and Figure 7). Furthermore, consistent with the gene expression data, lysates prepared from PRF and UBC decreased IL-6 protein levels in RAW264.7 cells (Figure 6D and Figure 7D). Together, these data showed that lysates extracted from PRF or UBC could reduce inflammation induced by TLR agonists.

### 2.6. Lysate from PRF and UBC Suppressed Nuclear Translocation of p65 in RAW264.7 Cells

**Figure 8:**
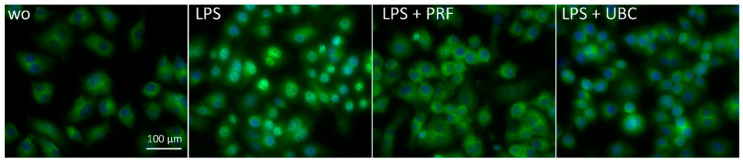
Lysates from PRF and UBC suppress the nuclear translocation of p65 in RAW264.7 cells. RAW264.7 cells were exposed to LPS in the presence or absence of lysates extracted from PRF or UBC. Immunofluorescence analysis of the nuclear translocation of p65 is shown. The blue dots represent nuclei which is hidden by the green dots representing antibody-positive nuclei; “wo” indicates without and represents unstimulated cells. Microscopic magnificence was set at 20×.

To further evaluate the inhibitory effect of PRF and UBC on NF-κB signaling, we carried out immunofluorescent analysis of NF-κB nuclear translocation. We observed a clear reduction in p65 nuclear staining induced by LPS in the presence of PRF and UBC (Figure 8 and Supplementary Figure S1). Overall, these data suggest that the anti-inflammatory activity of PRF and UBC is associated with a reduction in p65 nuclear translocation.

## 3. Discussion

PRF originating from coagulated fractionated blood is widely used in clinics not only because of the accumulating clinical evidence supporting its use but also because of the easily accessible equipment and preparation protocol of an autologous bioactive matrix with low costs [25]. However, it is unclear where there is a need to fractionate blood for the production of PRF if natural wound healing works with an ordinary blood clot [26]. There are more platelets and leukocytes in the fractionated plasma of PRF than in the unfractionated blood clot (UBC), but does this culminate in higher biological activity [27]? Current PRF research has not implemented UBC as an adequate control in various in vitro studies, and changes in biomechanical properties of the membranes originating from coagulated plasma alone or unfractionated blood have not been investigated. Based on this basic question, we have obtained three main findings.

The first main finding was that UBC is not a source of clinically stable membranes. The pseudo UBC membranes lack the integrity of PRF and appear to be dark red fragments. These findings support what is known about erythrocytes in that they hinder proper fibrin scaffold formation [22]. The viscoelasticity of plasma clots without erythrocytes was significantly increased relative to that of clots with erythrocytes [22]. However, we did not expect such obvious and pronounced reductions in biomechanical properties, making it impossible to prepare membranes from compressed UBC. Future research could investigate other strategies to prepare membranes from UBC, including lyophilization [28,29,30], the addition of aprotinin [31], and/or lysis of erythrocytes [32]. These strategies might not be clinically relevant for routine use in preparing membranes from UBC. However, our study used the fragments from the compressed UBC and PRF membranes for further bioassays. Thus, the membranes prepared from UBC disintegrated immediately after being squeezed with the compression plate/tray. Therefore, while PRF membranes were produced in this study, no UBC membranes could be obtained.

What remains to be determined is the role of erythrocytes in wound healing and bone regeneration. There is an incomplete understanding of the molecular details of erythrocyte clearance [33]. It may be that this step causes a delay in wound healing and bone regeneration or is an evolutionary trick to support would healing. Theoretically, erythrocytes could be space fillers in a blood clot, since their main purpose is oxygen transport [34]. However, erythrocytes are a rich source of catalase that acts as an antioxidant [35] and hemoglobin, which can reduce osteoclastogenesis [9,36], and these cells can act as damage-associated molecular patterns to modify innate immune responses [37]. Hemoglobin can restore cell proliferation via iron supplementation [38]. Thus, erythrocytes may not be passive bystanders in regenerative processes. We must be aware that when we prepare PRF, we generate biomechanically stable membranes, but we lack the possible effects of erythrocytes on wound healing and bone regeneration. Thus, future research might consider a possible beneficial effect of erythrocytes when preparing PRF, but not at levels affecting the integrity of the membranes.

Considering that this was the first study comparing PRF and UBC, there are limitations to acknowledge. Firstly, we used murine macrophage cell lines based on our previous established models [11,39]. However, applying human cell lines could be helpful to simulate in vivo status. Another limitation might be that the impact of erythrocytes to affect the weight of the clot is not standardized; thus, we must carefully interpret the normalization with respect to weight per volume. It may be that future studies use fibrinogen as a tool for normalization, as we have shown that there is a gradient when preparing PRF [40]. Questions may arise about the molecular mechanisms causing anti-inflammatory activity of PRF and UBC which are to some extend mediated by TGF-β [11]. However, this research is restricted to the present comparison of PRF and UBC based on our established bioassays [9,11,13]. 

Together, these in vitro findings suggest that there is not a significant difference in the TGF-β and anti-inflammatory activities of lysates extracted from PRF and UBC membranes. However, in contrast to PRF, compressed UBC failed to form clinically relevant membranes. Therefore, the centrifugation of blood remains a pivotal step that allows for the formation of clinically applicable fibrin-rich membranes. This research emphasizes that PRF and UBC share biological activity but not the biomechanical properties of PRF-derived membranes. It also signifies that the blood clot could be used as a control in future research for in vitro purposes.

## 4. Materials and Methods

### 4.1. Preparation of Lysates from PRF Membranes and Whole Blood Clots

PRF was prepared after acquiring the approval of the Ethics Committee of the Medical University of Vienna (1644/2018), and volunteers signed informed consent forms. All experiments were performed in accordance with relevant guidelines and regulations. To prepare lysates of the PRF membrane, venous blood was collected at the University Clinic of Dentistry from six healthy volunteers in plain glass tubes (Bio-PRF, Venice, FL, USA) and centrifuged at 700× *g* for 8 min (Z 306, Hermle, Universal Centrifuge, Wehingen, Germany) with universal swing-out rotors (146 mm at the max). The PRF clot was separated from the remaining red thrombus and compressed by the compression tray lid. For blood clot preparation, whole blood was left for half an hour to coagulate in the same tubes. PRF and clot membranes were transferred to serum-free medium (50 mg membrane/1.0 mL) and exposed to two cycles of freeze–thawing and sonication (Sonopuls 2000.2, BANDELIN Electronic, Berlin, Germany). The lysate was subjected to centrifugation at 15,000× *g* for 10 min, and the supernatant was filter sterilized and stored at −20 °C before in vitro analysis.

### 4.2. Cell Culture

Human gingiva was harvested from extracted wisdom teeth from patients who had given informed and written consent. Approval was obtained from the Ethics Committee of the Medical University of Vienna (EK NR 631/2007). A total of three strains of fibroblasts were established by explant cultures, and fewer than 10 passages were used for the experiments. Gingival fibroblasts and RAW264.7 macrophage-like cells (LGC Standards, Wesel, Germany) were grown and supplemented with 1% antibiotics (Sigma–Aldrich, St. Louis, MO, USA) and 10% fetal calf serum (Bio&Sell GmbH, Nuremberg, Germany). The cells were seeded at 30,000 cells/cm^2^ onto culture dishes one day prior to being stimulated. Cells were exposed to 30% PRF and UBC lysates for required time (overnight for the gene and protein expression, 1 h for the immunofluorescence experiment, 30 min for the WB analysis) under standard conditions at 37 °C, 5% CO_2_ and 95% humidity. Cells were treated with 10 ng/mL TGF-β1 (Cell Signaling Technology Europe, B.V., Frankfurt am Main, Germany) or 100 ng/mL LPS from *Escherichia coli* 0111: B41 (Sigma Aldrich, St. Louis, MO, USA), 10 µg/mL poly (1:C) HMW (InvivoGen, Toulouse, France), and 10 µg/mL FSL-1 (InvivoGen, Toulouse, France) in the indicated experiments. After overnight treatments (~16 h), gene and protein modulation were analyzed.

### 4.3. Reverse Transcription Quantitative Real-Time PCR (RT–qPCR) and Immunoassays

For RT–qPCR [41], after overnight stimulation, total RNA was isolated with the ExtractMe total RNA kit (Blirt S.A., Gdańsk, Poland) followed by reverse transcription (LabQ, Labconsulting, Vienna, Austria) and polymerase chain reaction (LabQ, Labconsulting, Vienna, Austria) on a CFX Connect™ Real-Time PCR Detection System (Bio–Rad Laboratories, Hercules, CA, USA). The primer sequences were as follows: mIL-6-F: GCTACCAAACTGGATATAATCAGGA, mIL-6-R: CCAGGTAGCTATGGTACTCCAGAA; mCOX-2-F: CAGACAACATAAAACTGCGCCTT, mCOX-2-R: GATACACCTCTCCACCAATGACC; miNOS-F: GGTGAAGGGACTGAGCTGTT, miNOS-R: ACGTTCTCCGTTCTCTTGCAG; mGAPDH-F: AACTTTGGCATTGTGGAAGG, mGAPDH-R: GGATGCAGGGATGATGTTCT; hIL-11-F: AAATAAGGCACAGATGCC, hIL-11-R: CCTTCCAAAGCCAGATC; hNOX-4a-F TCTTGGCTTACCTCCGAGGA; hNOX-4a-R: CTCCTGGTTCTCCTGCTTGG; hPRG4-F: CAGTTGCAGGTGGCATCTC, and hPRG4-R: TCGTGATTCAGCAAGTTTCATC. The mRNA levels were calculated by normalizing target gene expression to the housekeeping gene GAPDH using the ΔΔCt method. Supernatant levels of IL-6 were analyzed by immunoassays according to the manufacturer’s instructions (R&D Systems, Minneapolis, MN, USA). RT–PCR data are compared to the untreated control, which was considered 1.0 in all measurements so there was no need to show it as a separate group. However, in human IL-11 and murine IL-6 ELISA analyses, the absolute amount of secreted protein from the cells was reported, and untreated cells were also examined to show the amount of protein in all samples and compare the protein concentration.

### 4.4. Immunofluorescence Analysis

Immunofluorescence analysis of Smad2/3 and p65 nuclear translocation was performed in RAW264.7 cells and gingival fibroblasts, respectively. The cells were plated on Millicell^®^ EZ slides (Merck KGaA, Darmstadt, Germany) at a density of 15,000 cells/cm^2^. Cells were subjected to 30% lysates from PRF and UBC for one hour following overnight serum starvation. To induce inflammation, the cells were exposed to LPS from *Escherichia coli* 0111:B41 (Sigma–Aldrich, St. Louis, MO, USA) for 40 min. The cells were fixed with 4% paraformaldehyde, blocked with 1% bovine serum albumin (Sigma–Aldrich, St. Louis, MO, USA) and permeabilized with 0.3% Triton X-100 (Sigma–Aldrich, St. Louis, MO, USA). We used Smad2/3 (1:800; D7G7 XP^®^, Cell Signaling, MA, USA, #8685) and NF-κB p65 antibodies (IgG, 1:400, Cell Signaling Technology, #8242) at 4 °C overnight. Detection was performed with a goat anti-rabbit Alexa 488 secondary antibody (CS-4412, 1:800, Cell Signaling Technology). We captured the images on a fluorescence microscope with the DAPI-FITC dual excitation filter block (Echo Revolve fluorescence microscope, San Diego, CA, USA).

### 4.5. Western Blotting

Gingival fibroblasts were seeded at a density of 50,000 cells/cm^2^ in 6-well plates and serum-starved overnight. The following day, the cells were exposed to 30% lysates from PRF and UBC for 30 min. Extracts containing SDS buffer with protease and phosphatase inhibitors (Roche, Mannheim, Germany) were separated by SDS–PAGE and transferred to PVDF membranes (Roche Diagnostics, Mannheim, Germany). The membranes were blocked, and the binding of the rabbit phospho S423 + S425 (p-Smad3), (EP823Y, 1:1000, Abcam, Cambridge, UK), Smad3 (CS-9513, 1:1000, Cell Signaling Technology) and actin (sc-47778, 1:1000, Santa Cruz Biotechnology, SCBT, Santa Cruz, CA, USA) primary antibodies was detected with the HRP-labeled secondary antibody (CS-7074, anti-rabbit IgG, and CS-7076, anti-mouse IgG, both 1:10,000, Cell Signaling Technology). After exposure to the Clarity Western ECL Substrate (Bio–Rad Laboratories, Inc., Hercules, CA, USA), chemiluminescence signals were visualized with a ChemiDoc imaging system (Bio–Rad Laboratories). For densitometric analysis, images were analyzed using Image Lab software (Bio–Rad Laboratories).

### 4.6. Statistical Analysis

All experiments were performed four times. Each data point is representative of an independent experiment, which was individually obtained from a different blood donor in the treatment groups. Statistical analysis of IL-6 expression and immunoassays was performed with the Friedman test for multiple comparisons and the paired *t* tests for single comparisons. All groups were compared with the LPS, poly (1:C) HMW and FSL-1 groups as the positive control in the respective experiments. Analyses were performed using Prism v8 (GraphPad Software, La Jolla, CA, USA). Significance was set at *p* < 0.05.

## Data Availability

The original contributions presented in the study are included in the article/supplementary material. Further inquiries can be directed to the corresponding author.

## Supplementary Materials

The following supporting information is available online at https://www.mdpi.com/article/10.3390/ijms23115897/s1.
