# Supplementary material for: Blood Clots versus PRF: Activating TGF-β Signaling and Inhibiting Inflammation In Vitro
Source: Int J Mol Sci. 2022 May 24;23(11):5897. doi: 10.3390/ijms23115897 (PMC9180540; doi:10.3390/ijms23115897)
Supplement: Supplementary file 1 [file ijms-23-05897-s001.zip › ijms-1696575-supplementary.pdf]

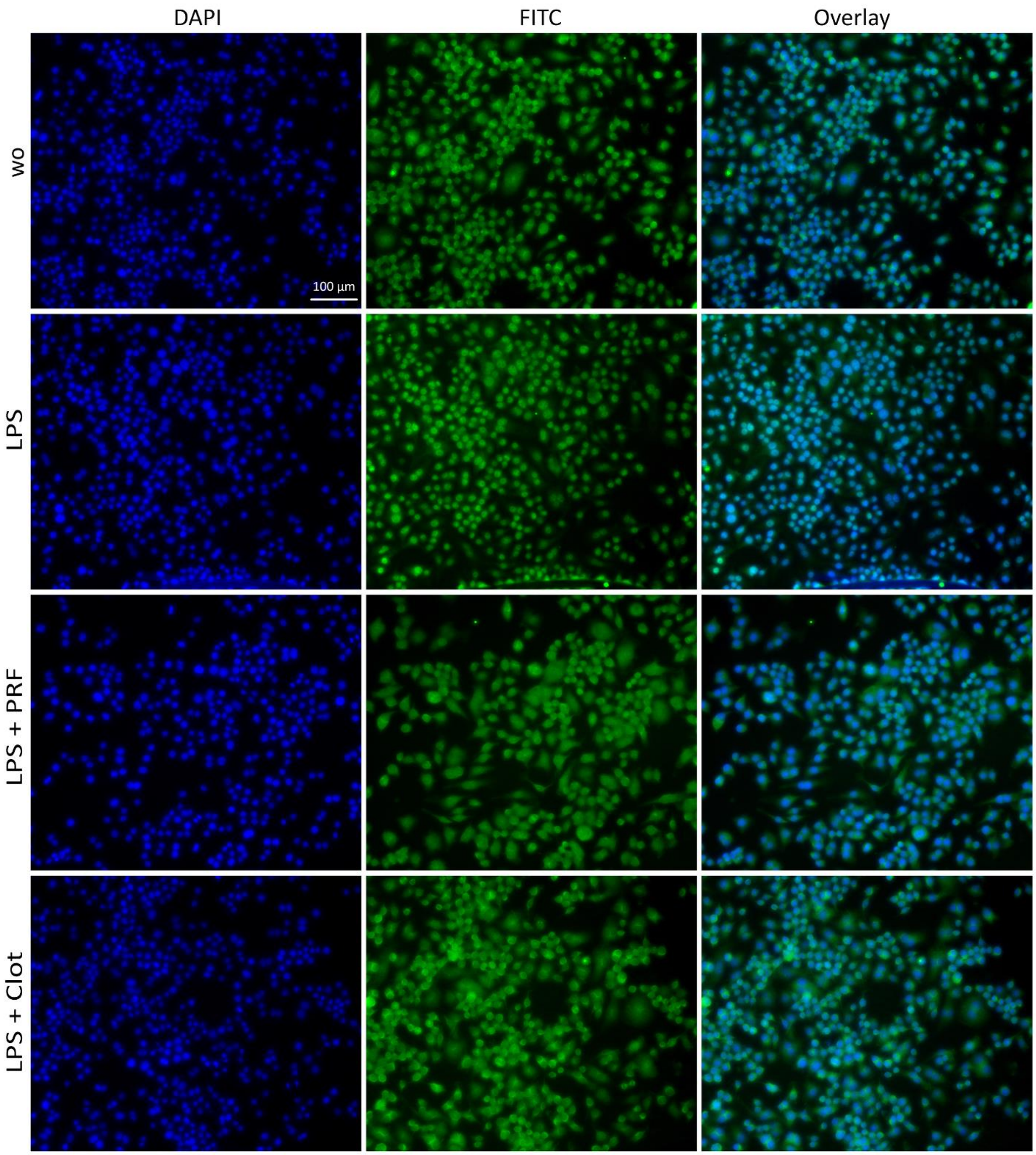

Figure S1: Lysates from PRF and UBC suppress the nuclear translocation of p65 in RAW264.7 cells. RAW264.7 cells were exposed to LPS in the presence or absence of lysates extracted from PRF or UBC. Immunofluorescence analysis of the nuclear translocation of p65 is shown using DAPI (blue) and FITC (green) filters. The blue dots represent nuclei which is hidden by the green dots representing antibody-positive nuclei in the overlay pictures; “wo” indicates without and represents unstimulated cells. Microscopic magnificence was set at 20X.
